# Scarring hidradenitis suppurativa with phrynoderma-like eruption, alopecia, and elevated interleukin 18

**DOI:** 10.1016/j.jdcr.2024.08.034

**Published:** 2024-11-19

**Authors:** Frank Z. Jing, Mika Yamanaka-Takaichi, Takashi K. Satoh, Kahlen R. Darr, Lars E. French, Afsaneh Alavi

**Affiliations:** a Department of Dermatology, Mayo Clinic, Rochester, Minnesota; b Department of Dermatology, Graduate School of Medicine, Osaka Metropolitan University, Osaka, Japan; c Department of Dermatology and Allergy, University Hospital, Ludwig Maximilian University (LMU) Munich, Munich, Germany; d Department of Genetics, Mayo Clinic, Rochester, Minnesota; e Dr. Phillip Frost Department of Dermatology and Cutaneous Surgery, University of Miami Miller School of Medicine, Miami, Florida

**Keywords:** genetics, genome sequencing, hidradenitis suppurativa, interleukin 18, NanoString, phrynoderma-like, scarring, skin of color

## Background

Interleukin (IL)-18 is related to the IL-1 family in terms of both structure and function, signaling through the IL-18 receptor complex that is found within normal human epidermis.^1^ Although IL-18 is constitutively expressed at low levels by human keratinocytes, the synthesis of biologically active IL-18 is dependent upon activation of caspase-1 and inflammasomes such as NLRP1 and NLRP3.^2^^,^^3^ IL-18 has demonstrated the ability to induce interferon gamma as well as stimulate T cells to produce other cytokines (eg, IL-2, granulocyte-macrophage colony-stimulating factor, and tumor necrosis factor-α) while suppressing IL-10.^4^ Its capability to induce different cytokines varies based on the target cell, showcasing a multifaceted role in immune responses. Here, we present a rare case of longstanding hidradenitis suppurativa (HS) with phrynoderma-like changes, extensive scarring, and markedly elevated IL-18.

## Case

A 46-year-old woman with a history of hypothyroidism and nutritional deficiency secondary to intensive dieting presented for management of longstanding HS. Her HS initially manifested as abscesses in the buttock area but quickly advanced to involve the axillae, groin, intergluteal folds, and inframammary regions. The disease ultimately led to extensive scarring across the trunk and extremities, as well as scarring alopecia of the scalp. This occurred despite conventional HS therapies including isotretinoin and a short course of ixekizumab. Adalimumab was also initiated but discontinued after 2 months after hospitalization because of sepsis.

**Fig 1:**
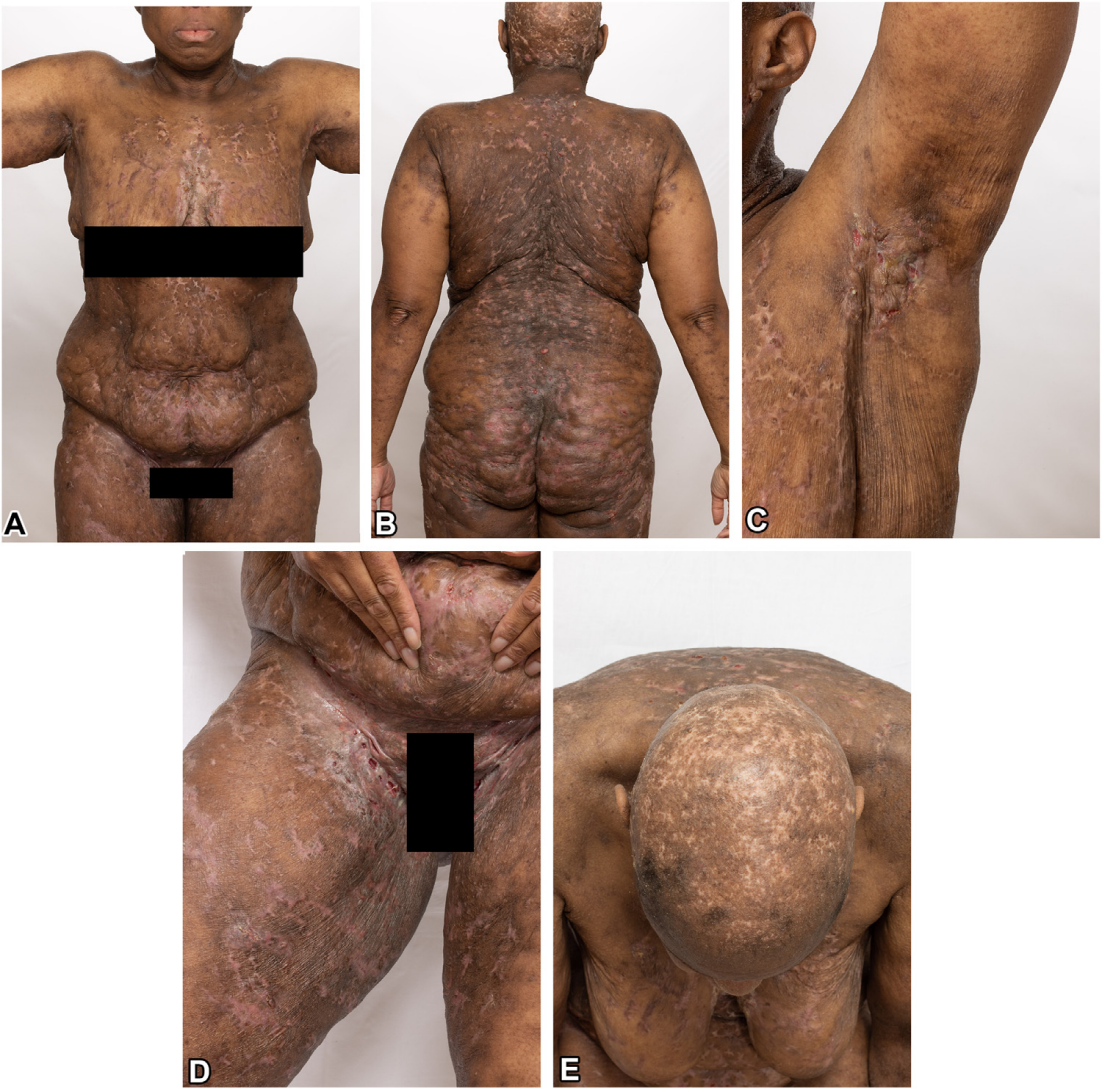
Clinical patient photographs of the front of body (**A**), back and buttocks (**B**), axilla (**C**), groin (**D**), and scalp (**E**).

**Fig 2:**
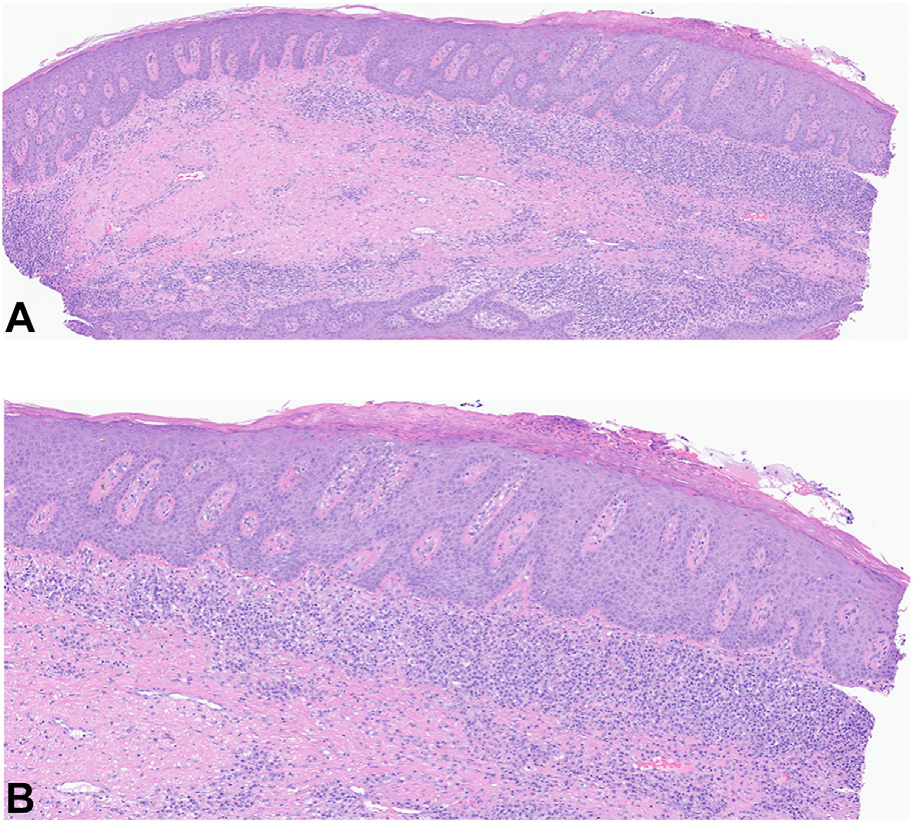
Hematoxylin and eosin stain of biopsy specimen obtained from left upper back. (Original magnifications: **A,** ×40; **B,** ×100.)

Her physical examination revealed phrynoderma-like lesions on the arms, trichodysplasia spinulosa-like lesions involving the nose, widespread reticular scarring, and knuckle pads raising suspicion of a genetic syndrome affecting the cornification process (Fig 1, *A-E*). Furthermore, the symptomatology raised the possibility of pyoderma gangrenosum-associated autoinflammatory syndromes. Biopsies at outside institutions 6 years before her presentation described papillated epidermal hyperplasia with follicular plugging and focal lichenoid dermatitis consistent with phrynoderma and follicular lichen planus, respectively. Repeat biopsy at our institution from 2 distinct morphologies revealed epidermal hyperplasia with parakeratosis, superficial papillary epidermal dyskeratosis, and papillary and reticular dermal and subcutaneous mixed inflammation, suggesting either a nutritional deficiency or eczematous dermatitis in the dermatitic lesions. The biopsy of the tunnels and nodules was consistent with HS (Fig 2, *A*, *B*). Repeat laboratory investigation at the time of repeat biopsy did not reveal any nutritional deficiencies.

Furthermore, her autoimmune work up was unrevealing. Antinuclear, double-stranded DNA, smith, ribonucleoprotein, Sjögren, scleroderma, and centromere antibodies returned negative. Infectious work up was equally unrevealing, with hepatitis B and C, QuantiFERON gold, HIV, and syphilis serologies returning negative multiple times throughout treatment course. However, our patient exhibited elevated inflammatory markers, including a c-reactive protein level of 5.4 mg/dL, an erythrocyte sedimentation rate of 96 mm/h, and leukocytosis as indicated by the complete blood count.

**Fig 3:**
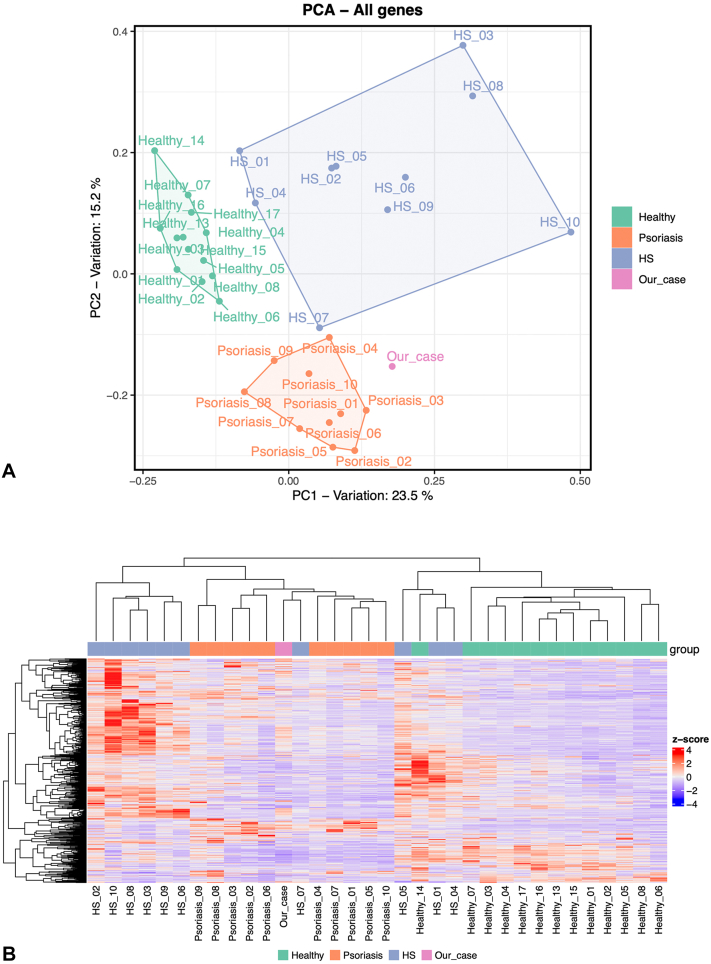
A, NanoString platform results displaying our patient’s tissue signature consistent with HS with overlapping characteristics of psoriasis and pyoderma gangrenosum. **B**, Gene expression heatmap displaying similarities between our patient’s and psoriasis patient samples.

**Fig 4:**
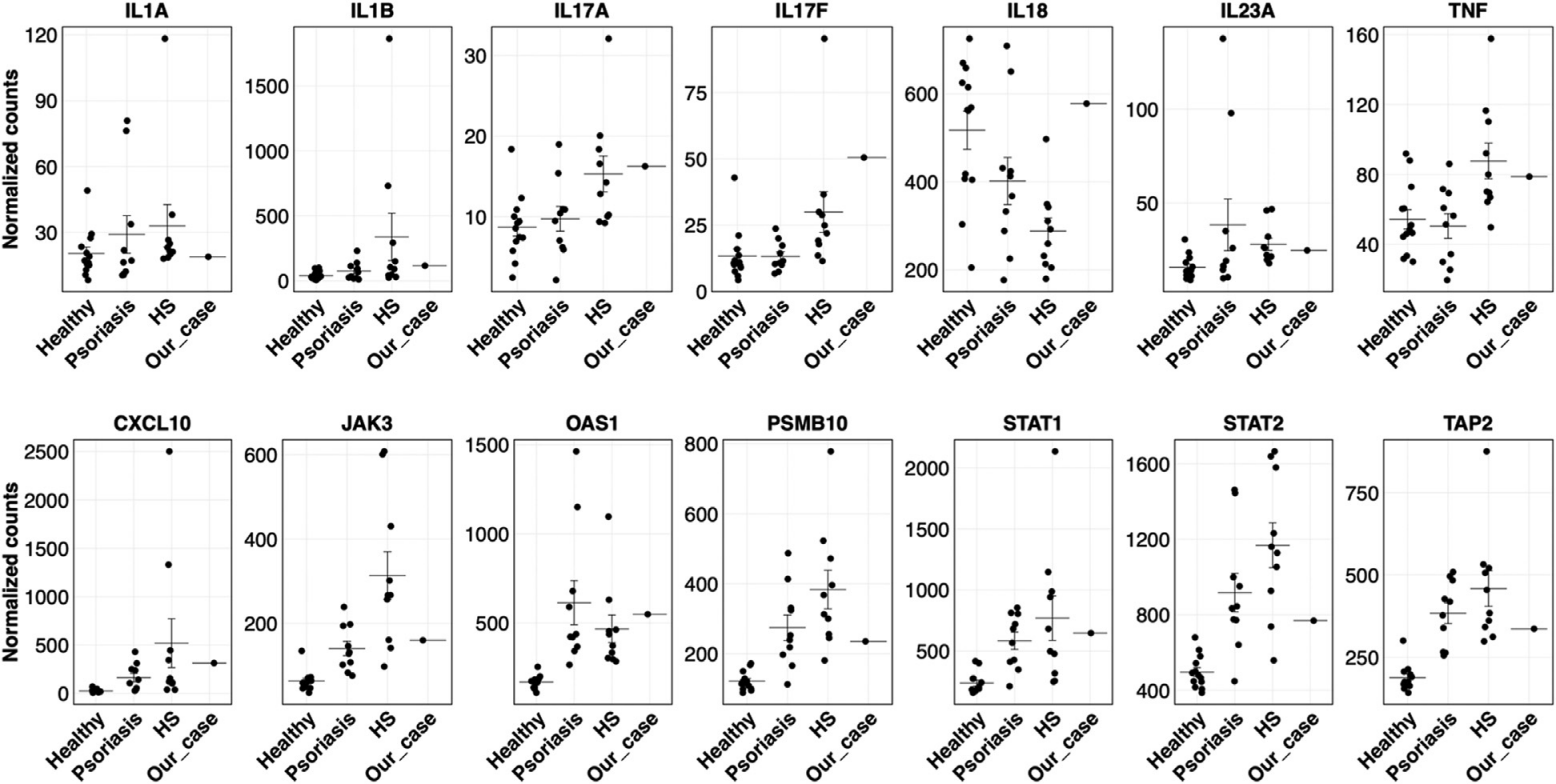
Normalized gene expression counts comparing our patient to healthy, psoriasis, and HS patients analyzed via NanoString platform.

An evaluation with cytokine panel demonstrated significant elevation of IL-18 of 8358 (upper limit of normal 468 pg/mL), IL-2 receptor α of 1473 (upper limit of normal 959 pg/mL), and IL-6. A repeat cytokine panel 3 months later redemonstrated elevated IL-18 of 3653 pg/mL and IL-6. NanoString platform testing revealed a tissue signature consistent with HS with overlapping characteristics of psoriasis and pyoderma gangrenosum (Fig 3, *A*). A gene expression heatmap revealed close clustering between the patient’s sample and psoriasis samples (Fig 3, *B*). Furthermore, the IL-18 in the patient sample was significantly elevated, along with IL-17A and IL-17F compared with HS and psoriasis samples previously analyzed. IL1A and IL1B expression did not differ significantly from healthy controls (Fig 4). Whole exome sequencing revealed a pathogenic variant of the filaggrin gene but failed to reveal any other abnormalities including mutations in PSTPIP1 or other known genes that would cause immunodeficiency. Given the elevated IL-17A and IL-17F and lack of expression of JAK-STAT pathways elucidated through NanoString analysis, treatment with bimekizumab was initiated. This resulted in substantial overall improvements and reduction in draining sinus tracts in follow up appointments.

## Discussion

IL-18 has been documented in its relation to a variety of dermatologic diseases (eg, psoriasis, rosacea). Kato et al^5^ reported a single nucleotide polymorphism in the IL-18 gene linked to psoriasis, indicating a role in psoriasis susceptibility through altering IL-18 production. This finding suggested that this single nucleotide polymorphism may have an important role in increasing susceptibility to psoriasis vulgaris by affecting the production of IL-18.^6^

HS is accepted as a manifestation secondary to immune dysregulation, however the precise pathogenesis is unknown. The current understanding involves elevated activity of dendritic and T cells, leading to the proliferation of keratinocytes through the influence of IL-23, IL-12, and the induction of a T helper 17 immune response. Kelly et al^7^ demonstrated IL-1β, IL-17, and active caspase-1 were enhanced in clinically normal perilesional skin and lesional skin. There was a significant increase in IL-18 expression within HS lesional skin and inhibition of caspase-1 led to a partial suppression of IL-18 expression.^7^ The identification of IL-18 is significant as it has been shown to promote the production of IL-17 by memory T cells, alongside IL-23.^8^ Furthermore, IL-18 may play a role in driving the activation of T helper 17 cells and the expression of IL-17.^8^

In our case, despite high IL-18 levels, a clear bias toward either the JAK-STAT or IL-1 pathways in the transcriptome was not observed, although increased expressions of IL-17A, IL-17F, IL-23A, and tumor necrosis factor-α were observed. Moreover, genes related to the interferon pathway, including CXCL10, JAK3, OAS1, PSMB10, STAT1/2, and TAP2, displayed heightened expression compared with healthy controls (see Fig 4). These findings suggest a potential benefit of using an anti-IL-17A and IL-17F, IL-23, or JAK inhibitors for future management. This aligns with recent studies describing successful management with these classes of medications.^9^, ^10^, ^11^ Further investigation into cytokine profiles in HS patients with recalcitrant disease should be considered as it may reveal other potential targets driving the disease process.

## Conflicts of interest

None disclosed.
